# Association of combined healthy lifestyle with general and abdominal obesity

**DOI:** 10.3389/fnut.2023.1332234

**Published:** 2024-01-16

**Authors:** Omid Sadeghi, Niloofar Eshaghian, Ammar Hassanzadeh Keshteli, Gholamreza Askari, Ahmad Esmaillzadeh, Peyman Adibi

**Affiliations:** ^1^Nutrition and Food Security Research Center, Department of Community Nutrition, School of Nutrition and Food Science, Isfahan University of Medical Sciences, Isfahan, Iran; ^2^Student Research Committee, Isfahan University of Medical Sciences, Isfahan, Iran; ^3^Department of Medicine, University of Alberta, Edmonton, AB, Canada; ^4^Obesity and Eating Habits Research Center, Endocrinology and Metabolism Molecular-Cellular Sciences Institute, Tehran University of Medical Sciences, Tehran, Iran; ^5^Department of Community Nutrition, School of Nutritional Sciences and Dietetics, Tehran University of Medical Sciences, Tehran, Iran; ^6^Integrative Functional Gastroenterology Research Center, Isfahan University of Medical Sciences, Isfahan, Iran

**Keywords:** lifestyle, diet, obesity, body mass index, waist circumference

## Abstract

**Background:**

Data linking joint healthy lifestyle factors to general and abdominal obesity are scarce, in particular in the Middle East. The aim of this study was to examine the association of combined healthy lifestyle factors with general and abdominal obesity in a large population of Iranian adults.

**Methods:**

This cross-sectional study was done on 3,172 Iranian adults aged ≥18 years. We constructed healthy lifestyle score using information on dietary intakes, physical activity, smoking status, and psychological distress. To evaluate components of healthy lifestyle, we applied a validated 106-item semi-quantitative Food Frequency Questionnaire (FFQ), General Practice Physical Activity Questionnaire (GPPAQ), General Health Questionnaire (GHQ), and other pre-tested questionnaires. General obesity was defined as having a body mass index (BMI) ≥30 kg/m^2^ and abdominal obesity as a waist circumference (WC) of ≥102 cm in men and ≥88 cm in women.

**Results:**

Mean age of participants was 36.54 ± 7.97 years. General and abdominal obesity were prevalent among 8.7% and 21.5% of study participants, respectively. Linear analysis showed a significant positive relationship between healthy lifestyle score and BMI among men (β: 0.30, 95% CI: 0.05, 0.54). However, no significant association was found between healthy lifestyle and abdominal obesity in men. Among women, one score increase in healthy lifestyle score was associated with a reduction of 0.65 cm in WC. In terms of individual components of healthy lifestyle, we found that low-distressed women had lower odds of abdominal obesity compared with high-distressed women.

**Conclusion:**

We found a significant inverse association between healthy lifestyle and WC among women. However, healthy lifestyle was positively associated with BMI among men.

## Introduction

Obesity has been an epidemic public health issue during the past decades which is associated with a greater risk of diabetes, hypertension, osteoarthritis, coronary heart disease (CHD), and some cancers (1–5). By 2030, it is projected that 20% of the worldwide population will be affected by obesity and 38% by overweight (6). In Iran, about half of the adult population are overweight or obese (7). In addition to general obesity, the prevalence of abdominal obesity has been estimated to be high (8). Compared with general obesity, abdominal obesity has been linked with a greater risk of mortality (9). Therefore, the assessment of determinants of general and abdominal obesity is of great importance.

Several modifiable risk factors including poor diet, sedentary lifestyle, smoking, and psychological distress have been assessed in relation to obesity (7, 8, 10–15); however, findings in this regard are conflicting. Healthy lifestyle was linked with a reduced risk of mortality (16). In a meta-analysis, a combination of at least four healthy lifestyle factors (obesity, alcohol consumption, smoking, diet, and physical activity) was associated with a 66% lower risk of all-cause mortality (16). Despite the assessment of individual lifestyle components, including diet, physical activity, or stress in relation to obesity, few studies have examined the association of combined lifestyle factors with general or abdominal obesity. Given the combined effect of these environmental factors on weight gain, it seems that the assessment of combined lifestyle factors can provide additional information on the incidence of this condition. On the other hand, it is not clear that the interaction between the mentioned lifestyle factors presented similar findings compared with individual factors in relation to obesity. In a cross-sectional study in Spain, a combination of four healthy lifestyle behaviors (including adherence to the Mediterranean diet, moderate alcohol consumption, expending ≥200 kcal/day in leisure-time physical activity, and being a non-smoker) was inversely associated with general and abdominal obesity (17). In a prospective cohort study, adherence to a healthy lifestyle [characterized by a healthy body mass index (BMI), high-quality diet, regular exercise, no smoking, and light to moderate alcohol intake] in mothers was associated with a substantially reduced risk of obesity in the children (18).

It must be kept in mind that all published studies so far were restricted to Western societies and few data are available from Asian countries, in particular from the understudied region of the Middle East. Assessing this association is particularly relevant for the Middle Eastern population, due to the high prevalence of a Middle Eastern pattern of obesity, which is characterized by abdominal fat accumulation and enlarged waist circumference (WC), particularly among women (19). In addition, lifestyle factors in the Middle East are different from other parts of the world. Because of cultural expectations, women in the Middle East have lower physical activity levels than men (20). In addition, smoking is more prevalent among men than women in this region (21). Furthermore, earlier studies on the association between healthy lifestyle and obesity have not considered stress as a major component of lifestyle. Therefore, the current study aimed to assess the link between the whole lifestyle factors and general and abdominal obesity among a large population of Iranian adults.

## Materials and methods

### Participants

This cross-sectional study was done on a large population of adults in Isfahan, Iran. Data of all participants were obtained from the SEPAHAN (Studying the Epidemiology of Psycho-Alimentary Health and Nutrition) project. Details on the study design, participants, and method of data collection were published previously (22). Briefly, this project was carried out in two separate phases. In the first phase, data on demographic variables and dietary intakes of participants, and in the second phase, data on mental health were collected. After merging the data from both phases of this project, 4,763 participants had complete data. In this study, we excluded participants with missing data on dietary intakes, demographic, anthropometric, and psychological data. We also excluded participants with an implausible energy intake (outside the range of 800–4,200 kcal/day). Moreover, pregnant and lactating women were not included in our study. Finally, 3,172 participants (1,398 men and 1,774 women) were included in the current analysis. Before starting the study, a written informed consent form was provided to all participants, and they were asked to sign it. The Bioethics Committee of Isfahan University of Medical Sciences, Isfahan, Iran, approved the SEPAHAN project (22).

### Assessment of dietary intakes

In the current study, information on participants’ dietary intakes was evaluated using a validated Willett-format Dish-based 106-item Semi-quantitative Food Frequency Questionnaire (DS-FFQ) (23). This questionnaire was specifically designed and validated for Iranian adults. Information on design, foods included, and the validity of this questionnaire was published elsewhere (23). We asked participants to report their dietary intakes based on nine multiple choice frequency response categories varying from “never or less than once a month” to “12 or more times per day.” The frequency response categories for the food list varied from six to nine choices. For foods with low consumption, we eliminated the high-frequency categories, while for foods with high consumption, we increased the number of multiple-choice categories. To convert the amount of each portion size to grams, we used the booklet of “household measures.” Daily intakes of foods and dishes were calculated according to the consumption frequency of each food item. Also, based on the nutrient contents of all foods and dishes, the daily nutrient intakes for each participant were computed. To obtain the nutrient contents of foods and dishes, we used the US Department of Agriculture’s (USDA) national nutrient databank (24).

To evaluate the validity and reliability of DS-FFQ, a subgroup of 200 participants of the SEPAHAN project was randomly selected (23, 25). All participants completed the DS-FFQ at baseline and 6 months later. During this 6 month, participants provided three dietary records. The findings showed that the DS-FFQ could provide valid and reliable measures of long-term dietary intakes in the Iranian population.

### Assessment of physical activity

By using the General Practice Physical Activity Questionnaire (GPPAQ), the physical activity of participants was evaluated (26). GPPAQ is a validated short instrument for the measurement of physical activity that was designed by the London School of Hygiene and Tropical Medicine (27). All participants were asked to report their activities based on this questionnaire. In this study, current physical activity has been used for the objective assessment of overall physical activity levels. The validity of GPPAQ for the assessment of habitual physical activity levels has earlier been shown (27).

### Assessment of psychological distress

Psychological distress was evaluated using the Iranian validated version of General Health Questionnaire (GHQ) (28). This questionnaire contains 12 items. Each item provides a 4-point rating scale (less than usual, no more than usual, rather more than usual, or much more than usual). By using the bimodal scoring method, we computed the total score of psychological distress for each participant (0-0-1-1). According to this method, total scores of GHQ range from 0 to 12; higher scores indicate a higher degree of psychological distress (29).

### Assessment of smoking

In the current study, a pre-tested questionnaire was used to evaluate the smoking status of participants. Participants were asked “How many cigarettes do you smoke every day?” They were able to choose one of the following options: “never smoked,” “I am an ex-smoker,” “1–5 cigarettes per day,” “5–20 cigarettes per day,” and “more than 20 cigarettes per day.” Participants were categorized into non-smokers, former smokers, or current smokers. In the present study, participants who reported smoking ≥1 cigarette per day were considered as current smokers.

### Construction of healthy lifestyle score

Diet, physical activity, smoking, and stress were considered to construct healthy lifestyle score. Healthy diet was defined based on Alternative Healthy Eating Index-2010 (AHEI-2010). To calculate AHEI-2010, we used a method designed by Kennedy et al. (30) and Sadeghi et al. (31). This method considered 11 components including fruit, vegetables, whole grains, nuts, and legumes, long-chain n-3 fats [docosahexaenoic acid (DHA) and eicosapentaenoic acid (EPA)], polyunsaturated fatty acid (PUFA), wine consumption, sugar-sweetened drinks and fruit juice, red and processed meats, trans-fat, and sodium intake. In the current study, due to a lack of information on wine consumption, 10 components were considered to compute AHEI. First, all study participants were classified into deciles according to the consumption of each component of AHEI-2010. Then participants in the highest decile of whole grains, vegetables, fruits, nuts, and legumes, long-chain n-3 fats (DHA and EPA), and PUFA received the score of 10, and those in the lowest decile were given the score of 1. Participants in other deciles of these food groups received the corresponding scores. In contrast, participants in the highest deciles of sugar-sweetened drinks and fruit juice, red and processed meats, trans-fat, and sodium received the score of 1, and those in the lowest deciles of these components were given the score of 10. Those in other deciles received the corresponding scores. By summing up the scores for these 10 components, we calculated the total HEI score for each participant. The total HEI score varied from 10 to 100. Participants in the highest 40% of AHEI (upper two-fifths) were considered as having a healthy diet.

With regards to physical activity, we classified participants into physically active (≥1 hour/week of moderate physical activity) and physically inactive (<1 hour/week of moderate physical activity). In terms of psychological distress, participants with a score of ≥4 were considered as having psychological distress. Regarding smoking, current smoking was considered as unhealthy behavior, and former smoking and non-smoking were considered as healthy.

**FIGURE 1:**
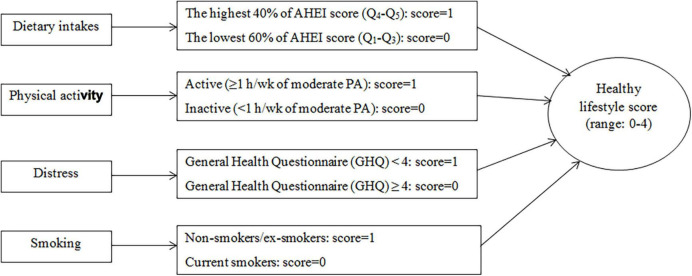
The healthy lifestyle score construction. AHEI, alternative healthy eating index; PA, physical activity; Q, quintile; h/wk, hour/week.

Finally, we calculated the total healthy lifestyle score by summing up the scores that each participant received for components of lifestyle. Subjects in the low-risk categories of the above-mentioned components (non/ex-smokers, those in the highest 40% of AHEI score, those with GHQ score of <4, and participants with ≥1 h/week of moderate physical activity) received the score of 1, otherwise, they received the score of 0. Therefore, a composite global healthy lifestyle score ranged from 0 to 4 (Figure 1). In the current study, the number of participants with a score of zero was very low. Therefore, we included participants with a score of 0 or 1 in one category as the score of 0/1 (the lowest category).

### Assessment of outcomes

Using a self-reported questionnaire, we gathered participants’ anthropometric information including height, weight, and WC. We computed BMI as weight in kilograms divided by the height in meters squared.

The validity of self-reported weight, height, and WC was evaluated in a pilot study on 200 participants from the same population. In the validation study, self-reported values of anthropometric indices were compared with actual measured values. The findings of this pilot study showed that the self-reported values of anthropometric indices provide a reasonable measure for these indices.

We defined obesity as having a BMI ≥30 kg/m^2^ and overweight as having a BMI ≥25 kg/m^2^. In the current study, we defined abdominal obesity based on WC according to the criteria proposed by the National Cholesterol Education Program (NCEP) (7, 32). Men with a WC of ≥102 cm and women with a WC of ≥88 cm, were considered as abdominally obese. We also defined abdominal overweight according to the criteria proposed by Lean et al. (33) as WC ≥94 cm for men and WC ≥80 cm for women.

### Assessment of other variables

To gather data on age, gender (male/female), marital status (single/married), family size (≤4/>4 members), breakfast skipping (yes/no) and house possession (owner/non-owner), diabetes (yes/no), use of anti-psychotic drugs (yes/no), and dietary supplements (yes/no), we used a self-administered questionnaire. Participants who were consuming breakfast <4 times/week were considered as breakfast skippers.

### Statistical analysis

We categorized participants according to the scores of healthy lifestyle (0/1 to 4). We included participants with a score of 0 or 1 in one category as the score of 0/1 (the lowest category). To assess differences across categories of healthy lifestyle scores, one-way analysis of variance (ANOVA) for continuous variables was used. In addition, Chi-square test was used to compare the distribution of individuals in terms of categorical variables across categories of healthy lifestyle score. To examine the association between healthy lifestyle score and general and abdominal obesity, we used binary logistic regression controlling for several covariates. Age, marital status (single/married), family size (≤4/>4 members), breakfast skipping (yes/no), house possession (owner/non-owner), history of diabetes (yes/no), use of anti-psychotic medications (yes/no), and dietary supplements (yes/no) were adjusted in the first model. For abdominal obesity, we also controlled for BMI in model 2 in addition to the variables included in model 1. In the highest versus lowest comparison, we considered participants in the lowest category of healthy lifestyle score as the reference group. To determine the trend of odds ratios (ORs) across increasing categories of healthy lifestyle score, we considered these categories as an ordinal variable. We also examined the dose-response association between each score increase in healthy lifestyle and general/abdominal obesity using binary logistic regression. The association between individual components of healthy lifestyle score and general and abdominal obesity was also examined in multivariable-adjusted models controlling for the above-mentioned covariates. We also compared the continuous indices of BMI and WC across the categories of healthy lifestyle scores using one-way analysis of covariance (ANCOVA). Moreover, to assess the linear association between healthy lifestyle scores and the mentioned indices, linear regression in an adjusted model was used. In the current study, we performed all statistical analyses in Statistical Package for Social Sciences (SPSS) software, version 18. *p*-Values of less than 0.05 were considered statistically significant.

## Results

In total, 3,172 individuals, with a mean age of 36.54 ± 7.97 years, were included in the current analysis. General and abdominal obesity were prevalent among 8.7% and 21.5% of study participants, respectively. General characteristics of men and women across categories of healthy lifestyle score are presented in Table 1. Compared with men in the lowest category of healthy lifestyle score, those in the highest category had higher BMI and greater adherence to AHEI and were less likely to be breakfast skippers, psychologically distressed, use dietary supplements and anti-psychotic medications, and were more likely to be house owner, physically active, and non/ex-smokers. Among women, those in the highest category of healthy lifestyle score had higher AHEI score and were less likely to be breakfast skippers, psychologically distressed, use anti-psychotic medications, and were more likely to be physically active and non/ex-smoker than women in the lowest category. No other significant differences were seen across categories of healthy lifestyle score in either gender.

**TABLE 1:** General characteristics of men and women across categories of healthy lifestyle score.

	Healthy lifestyle score				
	**1**	**2**	**3**	**4**	***p* ^a^**
Male					
*n*	216	608	464	110	
Age (year)	38.40 ± 7.72	37.90 ± 8.36	39.00 ± 8.11	39.00 ± 8.97	0.22
BMI (kg/m^2^)	24.86 ± 3.50	25.28 ± 3.49	25.68 ± 3.48	25.41 ± 2.86	0.04
Marital status (married) (%)	92.30	88.40	90.80	90.00	0.66
Obese^b^ (%)	6.30	9.20	10.10	4.80	0.20
Family size (>4 people) (%)	18.50	13.80	12.50	12.70	0.20
Breakfast skipper (%)	10.00	6.30	3.60	2.80	0.005
Diabetes (%)	4.20	2.00	3.20	3.60	0.32
Home ownership (owner) (%)	46.30	60.40	59.50	62.70	0.001
Dietary supplement use (%)	17.60	10.00	11.40	11.80	0.03
Anti-psychotic medications (%)	8.30	3.00	2.80	1.80	0.001
Physically active (≥1 h/week) (%)	1.40	7.10	33.20	100.00	<0.001
Smoking (non/ex-smoker) (%)	47.70	88.70	95.50	100.00	<0.001
Low levels of distress (%)	34.70	87.80	96.30	100.00	<0.001
High AHEI score (%)	3.70	16.40	75.00	100.00	<0.001
Female					
*n*	406	801	533	34	
Age (year)	34.70 ± 7.05	34.90 ± 7.56	35.90 ± 7.68	36.10 ± 7.47	0.06
BMI (kg/m^2^)	24.50 ± 4.12	24.37 ± 4.07	24.79 ± 4.02	23.77 ± 2.72	0.22
Marital status (married) (%)	75.80	73.30	71.80	69.70	0.81
Obese^b^ (%)	9.00	9.20	10.90	3.10	0.42
Family size (>4 people) (%)	14.50	11.70	11.40	8.80	0.41
Breakfast skipper (%)	12.20	9.20	6.00	3.10	0.01
Diabetes (%)	1.20	1.10	0.90	0.00	0.90
Home ownership (owner) (%)	58.90	60.40	59.80	61.80	0.70
Dietary supplement use (%)	44.60	41.70	42.00	47.10	0.74
Anti-psychotic medications (%)	13.10	5.50	6.20	5.90	<0.001
Physically active (≥1 h/week) (%)	1.00	3.20	11.10	100.00	<0.001
Smoking (non/ex-smoker) (%)	64.30	89.00	98.70	100.00	<0.001
Low levels of distress (%)	21.40	81.10	97.90	100.00	<0.001
High AHEI score (%)	4.20	26.60	92.30	100.00	<0.001

Data are presented as mean (± SD) or percent. BMI, body mass index; AHEI, alternative healthy eating index; h, hour; kg/m^2^, kilogram/square meter.

*^a^*Obtained from one-way analysis of variance (ANOVA) or Chi-square test, where appropriate.

*^b^*Defined as BMI of ≥30 kg/m^2^.

Dietary intakes of men and women across different levels of healthy lifestyle score are shown in Table 2. Compared with men in the lowest category of healthy lifestyle, those in the highest category had higher intakes of fruits, vegetables, fish, legumes plus nuts, whole grains, proteins, dietary fiber, vitamin B6, omega-3 fatty acids, and lower intakes of refined grains and caffeine. Among women, greater adherence to healthy lifestyle was associated with higher intakes of fruits, vegetables, fish, legumes plus nuts, whole grains, proteins, carbohydrates, dietary fiber, vitamin B6, omega-3 fatty acids, and lower intakes of refined grains, fats, red meat, and caffeine. No other significant difference was found.

**TABLE 2:** Dietary intakes of selected nutrients and food groups of men and women across different levels of healthy lifestyle score.

	Healthy lifestyle score				
	**1**	**2**	**3**	**4**	***p* ^a^**
Male					
Nutrients					
Energy (kcal)	2,526.67 ± 825.18	2,465.87 ± 863.48	2,468.22 ± 820.55	2,503.71 ± 815.90	0.80
Protein (g/d)	88.99 ± 14.95	89.02 ± 14.22	91.98 ± 14.63	92.09 ± 13.70	0.002
Fat (g/d)	102.50 ± 23.19	99.27 ± 19.87	99.54 ± 19.17	96.57 ± 19.00	0.07
CHO (g/d)	281.70 ± 59.84	289.54 ± 50.64	289.58 ± 51.99	297.49 ± 51.32	0.07
Fiber (g/d)	19.01 ± 4.62	20.46 ± 4.92	24.01 ± 5.37	26.17 ± 6.00	<0.001
Vitamin B6 (mg/d)	1.88 ± 0.43	1.89 ± 0.39	2.11 ± 0.40	2.15 ± 0.35	<0.001
Iron (mg/d)	17.78 ± 3.88	17.96 ± 3.63	17.66 ± 3.22	18.14 ± 3.32	0.41
Omega-3 FA (g/d)	1.70 ± 0.74	1.66 ± 0.52	1.83 ± 0.79	1.79 ± 0.58	<0.001
Caffeine (mg/d)	119.95 ± 104.06	99.04 ± 90.41	94.42 ± 79.44	89.95 ± 78.70	0.003
Food groups (g/d)					
Fruits	196.11 ± 163.53	219.51 ± 174.03	336.90 ± 226.24	381.80 ± 275.50	<0.001
Vegetables	194.78 ± 88.16	211.98 ± 108.28	258.59 ± 121.66	279.10 ± 102.71	<0.001
Red meat	86.44 ± 51.72	80.06 ± 40.10	81.84 ± 42.22	78.43 ± 35.58	0.24
Fish	7.85 ± 9.89	8.70 ± 11.14	13.45 ± 23.00	12.66 ± 12.89	<0.001
Legume plus nuts	42.73 ± 28.83	49.32 ± 38.69	64.14 ± 42.29	70.00 ± 39.62	<0.001
Whole grains	36.08 ± 65.33	34.38 ± 66.82	52.57 ± 86.51	60.92 ± 89.14	<0.001
Refined grains	422.23 ± 189.41	428.55 ± 169.83	358.08 ± 151.40	339.01 ± 160.26	<0.001
Dairy	308.71 ± 245.94	355.00 ± 275.60	334.13 ± 282.56	345.41 ± 284.46	0.18
Female					
Nutrients					
Energy (kcal)	2,268.40 ± 846.00	2,334.50 ± 800.93	2,298.21 ± 802.43	2,545.73 ± 797.19	0.19
Protein (g/d)	85.67 ± 13.46	87.43 ± 13.06	88.49 ± 13.35	88.09 ± 13.89	0.01
Fat (g/d)	100.45 ± 17.09	98.73 ± 17.43	96.90 ± 15.91	95.15 ± 13.31	0.01
CHO (g/d)	290.94 ± 43.25	294.70 ± 44.94	301.66 ± 44.49	307.61 ± 38.19	0.001
Fiber (g/d)	20.04 ± 4.82	22.23 ± 5.63	26.14 ± 5.47	27.29 ± 3.94	<0.001
Vitamin B6 (mg/d)	1.86 ± 0.37	1.94 ± 0.37	2.11 ± 0.36	2.16 ± 0.31	<0.001
Iron (mg/d)	17.34 ± 3.43	17.60 ± 2.99	17.32 ± 2.75	17.47 ± 2.26	0.32
Omega-3 FA (g/d)	1.71 ± 0.77	1.71 ± 0.68	1.86 ± 0.81	1.85 ± 0.71	0.002
Caffeine (mg/d)	109.50 ± 104.91	99.21 ± 84.23	92.13 ± 92.70	95.68 ± 80.75	0.04
Food groups (g/d)					
Fruits	249.30 ± 187.17	312.49 ± 199.02	472.74 ± 259.82	534.09 ± 207.52	<0.001
Vegetables	205.43 ± 99.02	235.60 ± 126.43	288.57 ± 113.91	306.01 ± 90.91	<0.001
Red meat	79.72 ± 37.02	79.16 ± 40.73	73.54 ± 36.34	68.74 ± 27.48	0.02
Fish	8.12 ± 10.49	9.85 ± 11.77	12.09 ± 12.50	13.08 ± 12.44	<0.001
Legume plus nuts	42.75 ± 26.00	47.06 ± 29.29	60.69 ± 33.22	64.74 ± 35.22	<0.001
Whole grains	36.24 ± 68.99	41.78 ± 77.10	48.96 ± 70.04	63.77 ± 75.32	0.02
Refined grains	417.04 ± 166.45	403.43 ± 162.02	343.96 ± 137.50	308.47 ± 137.36	<0.001
Dairy	350.57 ± 260.15	351.79 ± 248.90	344.33 ± 245.02	392.79 ± 328.91	0.73

Data are presented as mean (± SD). CHO, carbohydrate; FA, fatty acid; kcal, kilocalorie; g/d, gram/day; mg/d, milligram/day.

*^a^*Obtained from one-way analysis of variance (ANOVA).

Gender-stratified multivariable-adjusted means and standard errors (SE) and β-coefficients for the associations of healthy lifestyle score with BMI and WC in men and women are presented in Table 3. Among men, we found a significant difference in BMI across categories of healthy lifestyle score; however, after controlling for potential confounders, this difference became non-significant. Linear regression analysis showed a significant positive relationship between healthy lifestyle score and BMI among men so that in the fully adjusted model, one score increase in healthy lifestyle score was associated with a 0.3 kg/m^2^ increase in BMI. For WC among men, no significant association was found with healthy lifestyle scores. Among women, we found no significant association between healthy lifestyle score and BMI either based on the multivariable-adjusted means or based on β-coefficients for the linear association. However, for WC, after adjusting for confounding variables, one score increase in the healthy lifestyle score was associated with a reduction of 0.65 cm in WC.

**TABLE 3:** Gender-stratified multivariable-adjusted means (± SE) and β-coefficients for the associations of healthy lifestyle score with BMI and WC in men and women.

	Healthy lifestyle score					β for each score increase	
	**1**	**2**	**3**	**4**	***p* ^a^**	**β**	***p* ^b^**
Males							
BMI							
Crude	24.86 ± 0.25	25.28 ± 0.14	25.68 ± 0.16	25.41 ± 0.34	0.04	0.28 (0.05, 0.50)	0.02
Model 1	24.72 ± 0.27	25.24 ± 0.15	25.60 ± 0.18	25.42 ± 0.36	0.06	0.30 (0.05, 0.54)	0.02
WC							
Crude	90.73 ± 0.88	91.38 ± 0.48	91.95 ± 0.55	92.80 ± 1.15	0.45	0.64 (−0.14, 1.42)	0.11
Model 1	90.41 ± 0.97	91.30 ± 0.52	91.94 ± 0.59	92.47 ± 1.23	0.46	0.69 (−0.16, 1.53)	0.11
Model 2	91.75 ± 0.71	91.35 ± 0.38	91.41 ± 0.43	92.33 ± 0.90	0.76	0.11 (−0.51, 0.73)	0.72
Females							
BMI							
Crude	24.50 ± 0.20	24.37 ± 0.15	24.79 ± 0.18	23.77 ± 0.71	0.22	0.10 (−0.15, 0.35)	0.44
Model 1	24.62 ± 0.20	24.43 ± 0.14	24.69 ± 0.18	23.64 ± 0.69	0.36	−0.01 (−0.25, 0.24)	0.94
WC							
Crude	85.17 ± 0.62	84.40 ± 0.44	84.08 ± 0.54	81.68 ± 2.26	0.35	−0.63 (−1.39, 0.13)	0.10
Model 1	85.12 ± 0.62	84.48 ± 0.43	83.91 ± 0.54	81.00 ± 2.29	0.22	−0.73 (−1.49, 0.03)	0.06
Model 2	84.91 ± 0.47	84.67 ± 0.32	83.63 ± 0.41	83.58 ± 1.73	0.13	−0.65 (−1.22, −0.07)	0.03

Data are presented as means (± SE) or β (95% CI). BMI, body mass index; WC, waist circumference. Model 1: adjusted for age, marital status, family size, house possession, breakfast skipping, history of diabetes, current use of anti-psychotic drugs, and dietary supplements. Model 2: additionally, adjusted for BMI.

*^a^*Obtained from the one-way analysis of covariance (ANCOVA).

*^b^*Obtained from the linear regression.

Multivariable-adjusted OR and 95% confidence intervals (CIs) for the association of healthy lifestyle score with general and abdominal obesity are indicated in Table 4. Among men, after taking potential confounders into account, healthy lifestyle scores were not associated with overweight, obesity, and abdominal obesity (WC ≥102 or WC ≥94 cm). This non-significant association was also seen in the dose-response analysis based on one score increase in healthy lifestyle. Such non-significant associations were also observed among women.

**TABLE 4:** Gender-stratified multivariable odds ratios (ORs) and 95% confidence intervals (CIs) for general and abdominal obesity across different levels of healthy lifestyle score.

	Healthy lifestyle score				Per each score increase	
	**1**	**2**	**3**	**4**	**OR (95% CI)**	*p* *
Males						
BMI ≥30 kg/m^2^						
Cases (*n*)	12	53	44	5		
Crude	1.00	1.51 (0.79–2.90)	1.68 (0.87–3.26)	0.76 (0.26–2.21)	1.04 (0.82–1.31)	0.75
Model 1	1.00	1.33 (0.62–2.85)	1.53 (0.70–3.34)	0.53 (0.14–2.04)	0.99 (0.74–1.31)	0.93
BMI ≥25 kg/m^2^						
Cases (*n*)	102	303	253	60		
Crude	1.00	0.97 (0.70–1.35)	1.22 (0.87–1.72)	1.20 (0.74–1.95)	1.11 (0.97–1.27)	0.12
Model 1	1.00	0.98 (0.67–1.43)	1.29 (0.86–1.92)	1.25 (0.72–2.17)	1.14 (0.98–1.33)	0.10
WC ≥102 cm						
Cases (*n*)	13	76	53	13		
Crude	1.00	1.92 (1.03–3.57)	1.70 (0.89–3.23)	1.86 (0.82–4.25)	1.12 (0.90–1.38)	0.31
Model 1	1.00	2.03 (1.01–4.06)	1.88 (0.92–3.84)	1.66 (0.65–4.23)	1.10 (0.87–1.39)	0.40
Model 2	1.00	1.76 (0.82–3.78)	1.28 (0.57–2.84)	1.47 (0.52–4.18)	1.00 (0.76–1.32)	1.00
WC ≥94 cm						
Cases (*n*)	58	187	159	33		
Crude	1.00	0.95 (0.65–1.40)	1.13 (0.76–1.69)	0.96 (0.55–1.69)	1.05 (0.90–1.22)	0.55
Model 1	1.00	1.01 (0.65–1.57)	1.16 (0.73–1.84)	0.93 (0.49–1.76)	1.03 (0.87–1.23)	0.71
Model 2	1.00	0.82 (0.48–1.41)	0.85 (0.48–1.48)	0.68 (0.32–1.46)	0.92 (0.75–1.14)	0.45
Females						
BMI ≥30 kg/m^2^						
Cases (*n*)	35	70	56	1		
Crude	1.00	1.03 (0.67–1.57)	1.23 (0.79–1.93)	0.33 (0.04–2.47)	1.06 (0.86–1.30)	0.60
Model 1	1.00	1.03 (0.65–1.64)	1.04 (0.63–1.71)	0.32 (0.04–2.48)	0.97 (0.76–1.22)	0.77
BMI ≥25 kg/m^2^						
Cases (*n*)	157	289	226	10		
Crude	1.00	0.91 (0.71–1.17)	1.16 (0.89–1.51)	0.67 (0.31–1.46)	1.05 (0.93–1.19)	0.42
Model 1	1.00	0.85 (0.63–1.13)	1.13 (0.83–1.55)	0.64 (0.27–1.54)	1.04 (0.90–1.21)	0.57
WC ≥88 cm						
Cases (*n*)	121	248	149	9		
Crude	1.00	1.05 (0.80–1.38)	0.86 (0.64–1.16)	0.96 (0.41–2.24)	0.93 (0.81–1.07)	0.31
Model 1	1.00	1.06 (0.78–1.45)	0.86 (0.61–1.20)	0.96 (0.36–2.59)	0.92 (0.79–1.09)	0.34
Model 2	1.00	1.23 (0.84–1.79)	0.76 (0.50–1.15)	1.87 (0.62–5.67)	0.90 (0.74–1.09)	0.29
WC ≥80 cm						
Cases (*n*)	224	436	297	16		
Crude	1.00	0.93 (0.70–1.24)	0.94 (0.69–1.27)	0.82 (0.35–1.93)	0.97 (0.84–1.11)	0.64
Model 1	1.00	1.00 (0.72–1.39)	0.98 (0.69–1.40)	0.71 (0.25–1.96)	0.97 (0.82–1.15)	0.74
Model 2	1.00	1.08 (0.74–1.58)	0.90 (0.59–1.36)	0.95 (0.30–2.99)	0.94 (0.78–1.15)	0.56

Data are presented as OR (95% CI). BMI, body mass index; WC, waist circumference; kg/m^2^, kilogram/square meter; cm, centimeter. Model 1: adjusted for age, marital status, family size, house possession, breakfast skipping, history of diabetes, current use of anti-psychotic drugs, and dietary supplements. Model 2: additionally, adjusted for BMI.

*Obtained from the binary logistic regression.

The associations between individual components of healthy lifestyle and general and abdominal obesity are shown in Table 5. After controlling for potential confounding variables, none of the components of healthy lifestyle was associated with overweight, obesity, and abdominal obesity in men. However, in women, non-smokers had 61% higher odds of abdominal obesity (WC ≥80 cm) compared with smokers (OR: 1.61, 95% CI: 1.03–2.50). In addition, participants who had a low level of psychological distress had 29% lower odds of abdominal obesity compared with those who had a high level of distress (OR: 0.71, 95% CI: 0.51–0.98). For other associations assessed among women, we found no significant association in multivariable-adjusted models.

**TABLE 5:** Gender-stratified multivariable odds ratios (ORs) and 95% confidence intervals (CIs) for general and abdominal obesity across different levels of individual components of healthy lifestyle score.

Components of healthy lifestyle score	BMI ≥30 kg/m^2^	BMI ≥25 kg/m^2^	WC ≥102 cm (males)/WC ≥88 cm (females)	WC ≥94 cm (males)/WC ≥80 cm (females)
Males				
Physically active vs. physically inactive				
Crude	0.92 (0.57–1.47)	1.04 (0.80–1.36)	0.94 (0.62–1.42)	0.93 (0.69–1.26)
Model 1	0.72 (0.39–1.31)	1.08 (0.80–1.46)	0.89 (0.56–1.43)	0.82 (0.58–1.15)
Model 2			0.87 (0.50–1.51)	0.72 (0.47–1.09)
Non-smokers vs. smokers				
Crude	0.78 (0.47–1.32)	1.00 (0.73–1.38)	0.92 (0.56–1.50)	1.04 (0.72–1.50)
Model 1	0.98 (0.51–1.90)	1.33 (0.91–1.94)	1.19 (0.66–2.13)	1.22 (0.79–1.87)
Model 2			0.93 (0.47–1.81)	1.02 (0.60–1.72)
Low levels of distress vs. high levels of distress				
Crude	1.56 (0.86–2.83)	1.02 (0.76–1.37)	1.47 (0.87–2.48)	0.96 (0.68–1.35)
Model 1	1.34 (0.66–2.73)	0.92 (0.65–1.31)	1.51 (0.84–2.72)	1.05 (0.71–1.57)
Model 2			1.44 (0.74–2.82)	0.96 (0.59–1.57)
Healthy diet vs. non-healthy diet				
Crude	1.17 (0.79–1.72)	1.33 (1.06–1.66)	1.21 (0.86–1.71)	1.20 (0.93–1.54)
Model 1	1.14 (0.72–1.82)	1.28 (0.99–1.66)	1.07 (0.73–1.58)	1.14 (0.85–1.52)
Model 2			0.89 (0.56–1.40)	1.00 (0.70–1.42)
Females				
Physically active vs. physically inactive				
Crude	0.80 (0.40–1.62)	0.83 (0.56–1.23)	1.09 (0.70–1.69)	0.91 (0.58–1.43)
Model 1	0.95 (0.46–1.97)	0.80 (0.50–1.29)	1.14 (0.68–1.90)	0.86 (0.50–1.49)
Model 2			1.50 (0.82–2.72)	0.89 (0.48–1.64)
Non-smokers vs. smokers				
Crude	0.97 (0.60–1.55)	0.80 (0.60–1.06)	0.85 (0.62–1.18)	0.87 (0.62–1.22)
Model 1	1.05 (0.62–1.77)	0.90 (0.65–1.25)	1.01 (0.70–1.44)	1.20 (0.82–1.76)
Model 2			1.25 (0.81–1.92)	1.61 (1.03–2.50)
Low levels of distress vs. high levels of distress				
Crude	0.99 (0.69–1.42)	1.02 (0.82–1.27)	0.84 (0.66–1.06)	0.93 (0.73–1.20)
Model 1	0.86 (0.58–1.28)	0.94 (0.73–1.21)	0.77 (0.59–1.01)	0.87 (0.65–1.16)
Model 2			0.71 (0.51–0.98)	0.80 (0.57–1.13)
Healthy diet vs. non-healthy diet				
Crude	1.14 (0.82–1.58)	1.24 (1.02–1.51)	1.00 (0.81–1.25)	1.04 (0.83–1.29)
Model 1	0.92 (0.64–1.31)	1.23 (0.98–1.54)	1.00 (0.78–1.27)	1.00 (0.78–1.29)
Model 2			0.89 (0.66–1.20)	0.92 (0.68–1.24)

Data are presented as OR (95% CI). BMI, body mass index; WC, waist circumference; kg/m^2^, kilogram/square meter; cm, centimeter. Model 1: adjustments for age, marital status, family size, house possession, breakfast skipping, history of diabetes, current use of anti-psychotic drugs, and dietary supplements. Model 2: additionally, adjusted for BMI.

## Discussion

In the current study, we found a significant positive association between healthy lifestyle and BMI among men. Also, we found a significant inverse association between healthy lifestyle and WC among women. However, when we did analyses on binary variables including general and abdominal obesity, these associations became non-significant. In terms of individual components of healthy lifestyle, we found that non-smokers had higher odds of abdominal obesity than smokers in women. Furthermore, low-distressed women had lower odds of abdominal obesity compared with high-distressed women. To the best of our knowledge, this study is the first to examine the association between healthy lifestyle and general and abdominal obesity in a Middle Eastern population.

It is well-known that obesity, especially abdominal obesity, is a main risk factor for several metabolic disorders including cardiovascular diseases (CVDs) and diabetes mellitus (34–36). In the current century, the prevalence of obesity is increasing (8). Moreover, it has become an epidemic in the Middle Eastern population; so that more than two-thirds of Middle Eastern women are affected (8). Several modifiable risk factors including poor diet, sedentary lifestyle, smoking, and psychological distress have long been known to contribute to the obesity epidemic (7, 8, 10–15); however, little attention has been laid on the combined effect of these environmental factors on general and abdominal obesity. In the current study, we found a significant positive association between healthy lifestyle and the odds of general obesity among men. Contrary to our findings, Shook et al. (37) reported that adherence to a multiple national healthy lifestyle was inversely associated with odds of obesity among US children. However, in the study of Shook et al., only physical activity, drinking water, and diet quality were considered as components of healthy lifestyle. In a cross-sectional study in Spain, a combination of four healthy lifestyle behaviors including adherence to the Mediterranean diet, moderate alcohol consumption, expending ≥200 kcal/day in leisure-time physical activity, and non-smoking was associated with a lower prevalence of general and abdominal obesity (17). In addition, some studies revealed a significant inverse association between healthy lifestyle and obesity-related disorders including metabolic syndrome (38), diabetes, and hypertension (39). The controversy between our findings and those obtained from the previous studies might be attributed to the different components of healthy lifestyle among the previous studies. For instance, none of the previous studies considered psychological distress as a component of healthy lifestyle. In addition, different cut-off points used to determine each component of healthy lifestyle might be other reason for the controversy. Also, adjustments for different confounders might be another reason for this controversy. Moreover, it should be noted that there is a specific pattern of general obesity in the Middle Eastern population which is a bit different from others (19). Another reason for the unreasonable association between healthy lifestyle and general obesity in the current study might be due to the cross-sectional design of our study. On the other hand, obese participants usually tend to adhere to a healthy lifestyle in order to weight loss. Therefore, our findings on the link between healthy lifestyle and general obesity should be considered with caution, and further studies are needed to reveal facts in this regard.

In this study, a significant inverse association was found between healthy lifestyle and abdominal obesity among women. In line with our findings, the SUN cohort study showed that participants in the highest category of healthy lifestyle score (7–9 points) had a significantly reduced risk of developing abdominal obesity compared with those in the lowest category (0–3 points) (38). Such an inverse association was reported in a cross-sectional study from Spain (17). Diet, physical activity, and other components of healthy lifestyle score may influence obesity through several different pathways. Adherence to a healthy diet, which is associated with high consumption of whole grain foods and dietary fiber, can explain the favorable association at least to some extent (40). In addition, having a poor diet, cigarette smoking, psychological distress, and a sedentary lifestyle are associated with increased serum levels of inflammatory biomarkers (41–44). Inflammation can stimulate the accumulation of fatty acids in the abdomen (45).

In terms of individual components of healthy lifestyle, we found that non-smokers had higher odds of abdominal obesity than smokers in women. In contrast, Cheng et al. (46) reported that smoking was significantly associated with higher WC. Also, in a cross-sectional study, Clair et al. (47) showed a positive association between cigarettes smoked per day and central fat accumulation. Such a positive relationship between smoking and central obesity was also shown by other researchers (48, 49). The difference between our findings and other studies might be due to the different pattern of abdominal obesity in the Middle East, named Middle Eastern abdominal obesity, which is characterized by abdominal fat accumulation and enlarged WC, particularly among women (19). Also, the use of different methods to assess abdominal fat and different adjustments in analyses are other reasons for the controversy. Moreover, since the design of our study was cross-sectional, outcome may occur before exposure. On the other hand, abdominal obese individuals try to have a healthy lifestyle and therefore, they may tend to quit smoking.

In the present study, a significant positive association was found between psychological distress and abdominal obesity among women. Such association was also reported in previous investigations (50, 51). Psychological distress can increase food eating (52). On the other hand, distressed people tend to eat a high amount of food for decreasing their distress. Also, increased levels of glucocorticoids in distressed subjects increase the accumulation of fat in the abdomen (52).

Our study has several strengths including the large sample size, careful assessment of confounding variables as well as dietary intakes, psychological distress, and physical activity with validated questionnaires. Unlike earlier studies in which a single lifestyle behavior was assessed in relation to obesity, we examined the combined lifestyle score in relation to general and abdominal obesity. However, some limitations should be considered for interpreting our findings. Due to the cross-sectional design of the study, causality cannot be inferred. It is likely that obese people change their lifestyle in an effort to combat their weight status. Future investigations are needed to have a better understanding of the direction of this association. Measurement errors in epidemiologic studies are inevitable, in particular when one intends to assess diet and physical activity. However, such errors would attenuate the true associations. We tried to control for several potential confounding variables associated with the exposures and outcomes; however, residual confounding in our study, as in all epidemiological studies, is unavoidable. In addition, we used a self-report questionnaire to collect information on weight, height, and WC rather than a valid measurement. It has been reported that obese and overweight subjects tend to over-report height and underreport weight compared to normal-weight subjects (53).

## Conclusion

In conclusion, we found a significant positive association between healthy lifestyle and BMI among men. Also, we found a significant inverse association between healthy lifestyle and WC among women. In terms of individual components of healthy lifestyle, we found that non-smokers had higher odds of abdominal obesity than smokers in women. In addition, low-distressed women had lower odds of abdominal obesity compared with high-distressed women. Further studies, in particular of prospective nature, are required to confirm our findings.

## Data availability statement

The raw data supporting the conclusions of this article will be made available by the authors, without undue reservation.

## Ethics statement

The studies involving humans were approved by the Bioethics Committee of Isfahan University of Medical Sciences, Isfahan, Iran. The studies were conducted in accordance with the local legislation and institutional requirements. Written informed consent was gathered from each participant.

## Author contributions

OS: Methodology, Formal analysis, Writing – original draft. NE: Formal analysis, Writing – review & editing. AK: Methodology, Conceptualization, Project administration, Writing – review & editing. GA: Methodology, Funding acquisition, Writing – review & editing. AE: Methodology, Supervision, Conceptualization, Formal analysis, Project administration, Writing – review & editing. PA: Methodology, Conceptualization, Project administration, Writing – review & editing.
